# Baclofen for the Treatment of Alcohol Use Disorder in Patients With Liver Cirrhosis: 10 Years After the First Evidence

**DOI:** 10.3389/fpsyt.2018.00474

**Published:** 2018-10-01

**Authors:** Carolina Mosoni, Tommaso Dionisi, Gabriele Angelo Vassallo, Antonio Mirijello, Claudia Tarli, Mariangela Antonelli, Luisa Sestito, Maria Margherita Rando, Alberto Tosoni, Salvatore De Cosmo, Antonio Gasbarrini, Giovanni Addolorato

**Affiliations:** ^1^“Alcohol Use Disorder and Related Disease” Unit, Department of Internal Medicine and Gastroenterology, Catholic University of Rome, Rome, Italy; ^2^Department of Internal Medicine and Gastroenterology, Catholic University of Rome, Rome, Italy; ^3^Emergency Department, V. Cervello Hospital, Palermo, Italy; ^4^Department of Medical Sciences, IRCCS Casa Sollievo della Sofferenza Hospital, San Giovanni Rotondo, Italy

**Keywords:** alcohol use disorder, baclofen, alcoholic liver disease, liver cirrhosis, GABA-B receptor

## Abstract

Alcohol Use Disorder (AUD) is a chronic and relapsing condition characterized by harmful alcohol intake and behavioral-cognitive changes. AUD is the most common cause of liver disease in the Western world. Alcohol abstinence is the cornerstone of therapy in alcoholic patients affected with liver disease. Medical recommendations, brief motivational interventions and psychosocial approach are essential pieces of the treatment for these patients; however, their efficacy alone may not be enough to achieve total alcohol abstinence. The addition of pharmacological treatment could improve clinical outcomes in AUD patients. Moreover, pharmacological treatments for AUD are limited in patients with advanced liver disease, since impaired liver function affects drugs metabolism and could increase the risk of drugs-related hepatotoxicity. At present, only baclofen has been tested in RCTs in patients with advanced liver disease. This medication was effective to reduce alcohol intake, to promote alcohol abstinence and to prevent relapse in AUD patients affected by liver cirrhosis. In addition, the drug showed a safe profile in these patients. In this review, clinical studies about efficacy and safety of baclofen administration in patients with AUD and advanced liver disease will be reviewed. Open question about the most appropriate dose of the drug, duration of the treatment and need of additional studies will also be discussed.

## Introduction

Alcohol Use Disorder (AUD) represents problematic patterns of alcohol consumption, leading to clinically significant impairment or distress (1). It is characterized by behavioral and cognitive changes as tolerance and craving for alcohol, and withdrawal syndrome at abrupt alcohol reduction or discontinuation.

AUD is responsible for over 2.5 million deaths every year in the world (2) and it represents the third leading risk factor for morbidity and mortality in Europe (3).

AUD represents a risk factor for alcoholic liver disease (ALD), ranging from steatosis and alcoholic hepatitis to liver cirrhosis and its complications (e.g., hepatocellular carcinoma). The risk of developing ALD increases with the amount of alcohol intake and its duration (4). Besides alcohol's direct toxicity, hepatitis virus co-infection, overweight and host factors (i.e., gut microbiota, gender, genetic, nutritional factors and comorbidities) are additional factors, influencing the development and the progression of liver disease (5, 6).

ALD causes yearly half a million deaths worldwide, accounting for 50% of global liver disease-related mortality (7). Moreover, ALD is one of the most common indication for liver transplantation (LT) in Europe and North America (6).

Given the strong relationship between alcohol discontinuation and hepatic function improvement, regardless the stages of liver disease, total alcohol abstinence represents the main outcome in the treatment of AUD patients with liver disease (7). Medical recommendations, brief motivational interventions and/or psychosocial approach alone, although essential components for AUD treatment, may not be sufficient to induce total alcohol abstinence and prevent relapse. The addition of effective pharmacological treatment for AUD may be very useful (8). However, given the impaired hepatic function and the lack of randomized clinical trials (RCTs) investigating both efficacy and safety of approved medications for AUD (disulfiram, naltrexone, nalmefene and acamprosate) in patients with liver disease, the availability of pharmacological treatments for AUD is limited for this group of patients (9).

Baclofen is a selective GABA-B receptor agonist with primary kidney metabolism. After promising results in preclinical model of alcohol abuse and clinical studies in AUD patients without liver disease [for review see (10)], it was tested in AUD patients with advanced liver disease, including patients affected with liver cirrhosis not complicated by hepatic encephalopathy nor hepato-renal syndrome. In the present review, clinical studies investigating baclofen administration in AUD patients with liver disease were analyzed. The analysis included RTCs, observational studies and case series published in English from December 8th 2007 to May 24th 2018 (Table S1). The studies were searched on PubMed using the words AUD, Baclofen, anti-craving drugs, pharmacotherapy for AUD, liver cirrhosis, alcoholic liver disease and through citation chaining. Case reports were excluded from this analysis.

## Baclofen in patients with liver disease

Efficacy and safety of baclofen in AUD patients affected by advanced liver disease were firstly tested in a double-blind, placebo-controlled clinical trial (RCT) (11). In this study, 84 AUD patients with liver cirrhosis were randomized to baclofen treatment (10 mg t.i.d.) or to placebo. The proportion of patients with total alcohol abstinence (71% of the patients who received baclofen, 29% of the patients allocated to placebo; odds ratio 6·3 [95% CI 2·4–16·1]; *p* = 0·0001) and cumulative abstinence duration (mean 62·8 [SE 5·4] in baclofen group vs. 30·8 [5·5] days in placebo group; *p* = 0·001) was significantly higher in the group treated with baclofen. No differences on side effects were found between the two groups. No new-onset episode of overt hepatic encephalopathy was reported, also considering subjects with severe hepatic impairment (Child-Pugh classes B and C). The total alcohol abstinence was particularly evident in patients with more advanced liver cirrhosis, as indicated by the Child-Pugh score. The odd-ratio to maintain abstinence compared to placebo exceeded 4 in child B group and 8 in Child C group. These data suggested a possible relationship between the efficacy of the drug and the severity of AUD (11).

In a *post-hoc* analysis of this study, 24 AUD patients with hepatitis C virus infection were included. In particular, 12 were allocated baclofen 10 mg t.i.d., while 12 received placebo. A significantly higher number of patients who achieved and maintained total alcohol abstinence was found in the baclofen group with respect to the other group. Considering that their baseline characteristic differed for blood level of transaminases probably due to HCV-related damage, albumin and INR were chosen as outcome measure. A significantly higher increase in albumin values from baseline (*p* = 0.0132) and a “trend toward a significant reduction in INR levels from baseline (*p* = 0.0716)” was observed in the baclofen group. These data firstly suggested that baclofen treatment may represent an optimal and safe anti-craving medication in this typology of patients (12).

Based on observation of a possible dose-dependent effect in case series (13–15) and in a*n* RCT (16), a subsequent retrospective study investigated a tailored-dose of baclofen in 53 patients with alcoholic liver disease, comparing alcohol consumption and hospitalizations before and after baclofen treatment (17). Median highest dose administered was 60 mg/d. A trend in the decrease of hospitalizations was found in patients after baclofen treatment (on average, after baclofen initiation the patients spent 19.1 days in hospital per year, compared with 25.84 days per year before treatment initiation; *p* = 0.59) coupled with a reduction of alcohol consumption. No improvement in patients' quality of life, depression and anxiety during hospitalizations was recorded. Baclofen treatment was generally well tolerated, although a dose reduction was necessary in four patients. The strength of this study is the real-life experience, although the small sample size, the amount of missing data, the retrospective design and the absence of a control group limit its results. In this latter study, the relationship between severity of liver disease (Child score for cirrhotic patients) and maximum dose of baclofen used, although not significant, suggested that patients with high severity of liver disease might require lower doses of baclofen to suppress craving. It is conceivable that the small percentage of the drug metabolized in the liver (about 15%) is not metabolized in patients with severe liver dysfunction, increasing in these patients the blood level of medication (18). This observation could also be consistent with the higher efficacy of baclofen in patients with more severity of liver disease reported in the first RCT (11).

In a subsequent prospective cohort study (19), 219 consecutive patients with ALD (including also patients with liver cirrhosis) were treated with dose titration of baclofen up to 30 mg t.i.d according to tolerability and response to the drug. Although the lack of a control group and the observational nature represented important limits of this study, baclofen administration had a positive impact on measures of alcohol consumption and adherence to treatment was very high. Moreover, the strength point of this study was the real-life experience in clinical practice in a joint liver and alcohol treatment clinic.

A recent prospective study conducted by Barrault et al. (20) showed a significant decrease in alcohol consumption in 100 AUD patients, 65 of them affected by liver cirrhosis, after treatment with tailored dose of baclofen (mean dosage 40 mg/d; range 30–210 mg/d). Patients were recruited over a 3-year period and they were followed for one-year in two liver and alcohol outpatient clinics. A marked improvement in liver function tests was found in patients who discontinued alcohol drinking with respect to patients who did not respond to baclofen treatment and continued alcohol consumption (20). No drug-related serious adverse events occurred, no hepatic encephalopathy, liver function and/or renal impairment were detected in treated patients. Minor side effects, such as drowsiness and vertigo were found; these symptoms decreased after tapering the dose (20). No evidence of baclofen abuse or overdose was identified. No baclofen withdrawal syndrome was observed in patients who stopped baclofen suddenly. The long-term duration of follow-up (1 year) represents a strength point of this study, although main limitation was the absence of a control group.

Two RCTs with contrasting results were recently published (21, 22). In the first one a total of 180 US veterans were enrolled (22). These patients were affected by AUD and chronic hepatitis C virus infection with ongoing alcohol consumption. Patients were randomized to baclofen treatment (30 mg/d) or placebo for 12 weeks. The primary outcome was the difference of percentage of days of abstinence. Secondary outcomes were the percentage of patients who achieved complete alcohol abstinence, the percentage of heavy drinking days, alcohol craving, anxiety, depression and post-traumatic stress disorder. No differences between the two groups of treatment in term of percentage of abstinent days was reported. No significant difference in secondary outcomes was found between baclofen and placebo group. However, it should be underlined that Veterans represent a specific group of patients as the enrolled patients were also affected by psychiatric comorbidities and use of illicit drugs. These observations prevent to draft definitive conclusions and to generalize these results on the overall AUD population, although this is the largest RCT examining baclofen efficacy and safety on liver disease patients up to date.

The second one is a very recent multi-site, double-blind, randomized placebo-controlled clinical trial named BacALD (21), which investigated the efficacy and safety of 2 fixed dose of baclofen (30 mg/d and 75 mg/d). In this study, 104 patients with AUD were enrolled. Among them, 58 patients were affected by ALD. Primary outcomes included survival time to lapse and relapse, and the composite outcome of drinks per drinking day, number of heavy drinking days and percentage abstinent days. With respect to placebo, a significant efficacy of baclofen on time to lapse (χ^2^ = 6.44, *P* < 0.05, Cohen's *d* = 0.56) and to relapse (χ^2^ = 4.62, *P* < 0.05, *d* = 0.52) was found, with no difference between the 2 doses of the drug. Moreover, a significant increase in the number of days to first lapse and relapse was found in ALD subgroup of patients. Percentage of days of alcohol abstinence was significantly higher in baclofen group with respect to placebo group, with no difference between the 2 doses of the drug (placebo 43%, baclofen 30 mg 69%, baclofen 75 mg 65%; *P* < 0.05). Although the majority of patients showed a good tolerability for the drug, patients randomized to the 75 mg dose of baclofen reported significantly more sedation and shortness of breath compared with those randomized to the 30 mg dose. In conclusion, this study supports the efficacy and safety of baclofen in the treatment of AUD patients with ALD. Moreover, no reasons to promote the use of daily doses higher than 30 mg in these patients emerged.

## Baclofen in acute alcoholic hepatitis

Alcoholic hepatitis (AH) is a severe clinical syndrome characterized by the recent onset of jaundice with or without other signs of liver decompensation in patients with ongoing alcohol abuse. Its histological features consist of steatosis, hepatocyte ballooning, and inflammatory infiltrate with polymorphonuclear neutrophils. It is associated with a high rate of morbidity and mortality (22). Corticosteroid therapy should be considered in patients with severe AH, although this treatment could not influence medium to long term survival (9). Alcohol abstinence remains the cornerstone of therapy and early management of AUD is mandatory in all patients with AH (9). However, trials investigating the use of anti-craving drugs are currently lacking in these patients. Only a single center, open, retrospective study analyzed the effects of baclofen in patients with acute alcoholic hepatitis (23). In this study 35 patients were evaluated; baclofen treatment was started when bilirubin level decreased below 10 mg/dl and after hepatic encephalopathy resolution. 10 mg t.i.d. of baclofen was used, on average, for 5.8 months; of the 35 patients treated with baclofen, 34 (97%) remained abstinent. An improvement of liver function tests and a significant decrease of severity of liver disease expressed as MELD score was observed in all treated patients. Although the retrospective design and the lack of a control group represent significant limits of the study, these observations support the safety and the usefulness of the drug in improving the clinical condition of patients with ALD. Future RCT on this special population of AUD patients are needed to confirm its efficacy and safety.

## Baclofen in patients with alcohol withdrawal syndrome and liver disease

Alcohol withdrawal syndrome (AWS) is a potentially life-threatening medical condition developing in patients who abruptly cease or reduce alcohol consumption (24). At present, the gold standard therapy is represented by benzodiazepines in the management of moderate-severe forms of AWS, given their efficacy in controlling both withdrawal symptoms and the risk of seizures and/or delirium tremens. However, some benzodiazepines own hepatic metabolism, producing active metabolites that raise the risk of drug accumulation and excess of sedation in patients with advanced LD. Among BZDs, lorazepam or oxazepam may be preferred, given their shorter half-life and absence of active metabolites (24). Considering potential side-effects of benzodiazepines in patients with LD (25), non-benzodiazepine GABAergic drugs, might be useful in the management of AWS in patients with advanced liver disease, given their low rate of hepatic metabolism (7). Among them, Baclofen, based on its safety hepatic profile (11), seems to be a promising agent for the treatment of AWS in patients with LD, since its efficacy in the management of AWS in patients without LD has been showed (26–28). However, RCT data are required to validate the preliminary results on the use of these drugs in AWS, in particular in AUD patients with ALD.

## Conclusions

Although the role of baclofen in the treatment of AUD is still debated, the data available at moment suggest that the drug is effective and safe, in particular in some subset of patients, including those with high severity of AUD (29) and advanced liver disease (11, 21).

Additional RCTs are needed to clarify some drug aspects, in particular the most appropriate dosage and its role in AUD patients with different comorbidity.

Further trials are also required in AUD patients with ALD to compare baclofen to other anti-craving drugs, i.e., with Acamprosate, which showed a good tolerability in Child-Pugh stage A and B cirrhotic patients, although the available data are limited to a 1-day trial (30). Moreover, given the controversial results emerged about the efficacy of the drug in AUD patients affected by HCV infection (12, 31), this topic should be further investigated, considering the importance of alcohol discontinuation in HCV patients (32). Indeed, baclofen could have a potential role as bridge or concomitant treatment with antiviral therapy (12).

Finally, a further topic of interest is the potential role of baclofen in AUD patients listed for liver transplantation. At present liver transplantation represents the gold standard treatment for AUD patients affected by end-stage liver disease (33). In an era of organ shortage, it is mandatory to reduce the risk of alcohol relapse in these patients, in particular after transplantation in order to reduce the probability of graft loss and the liver damage, so total alcohol abstinence should be promoted not only before but also after LT. In view of the safety hepatic profile of baclofen, this drug could be the most appropriate medication to promote alcohol abstinence and to prevent relapse in AUD transplanted patients.

## Author contributions

CM, TD, GV, AM, CT, MA, LS, MR, AT, AG, and GA contributed equally to this work. SD helped the revision process of the paper, playing major role in answering to reviewers, table conceiving, modifying the manuscript and editing English language.

### Conflict of interest statement

The authors declare that the research was conducted in the absence of any commercial or financial relationships that could be construed as a potential conflict of interest.

## Supplementary material

Table S1 Main features of analyzed study.
