# Supplementary material for: Baclofen for the Treatment of Alcohol Use Disorder in Patients With Liver Cirrhosis: 10 Years After the First Evidence
Source: Front Psychiatry. 2018 Oct 1;9:474. doi: 10.3389/fpsyt.2018.00474 (PMC6174362; doi:10.3389/fpsyt.2018.00474)
Supplement: Table S1 — Main features of analyzed study. [file Data_Sheet_1.PDF]

Study	Type of study	Sample size (pz)	Dose (mg/d)	Length of the study (weeks)	Outcome	Results			p value
Addolorato et al. 2007 (11)	RCT	84	30	16	proportion of patients achieving and maintaining alcohol abstinence	placebo 29%	baclofen 71%		0.0001
Leggio et al. 2012 (12)	Post hoc analysis*	24	30	16	proportion of patients achieving and maintaining alcohol abstinence	placebo 25%	baclofen 83%		0.0123
Hauser et al. 2016 (31)	RCT	180	30	12	percentage of days of abstinence from alcohol	placebo 77%	baclofen 75%		0.95
Morley et al. 2018 (21)	RCT	104	30 and 75	12	percentage of days of abstinence from alcohol	placebo 43%	baclofen 30mg 69%	baclofen 75mg 65%	<0.05
Yamini et al. 2014 (23)	Cohort study, retrospective	35	30	52	proportion of patients achieving and maintaining alcohol abstinence	baclofen 97%			n.a.
Owens et al. 2016 (19)	Cohort study, prospective	219	90	13 and 52	proportion of patients achieving and maintaining alcohol abstinence	55% at week 13; 53% at week 52			n.a.
Barrault et al. 2017 (20)	Cohort study, prospective	100	40**	52	percentage of patients abstinent and of patients that reduced alcohol consumption	abstinent patients 44%; patients that reduced alcohol consumption (<30g/dl) 20%			n.a.
Heydtmann et al. 2015 (17)	Case series	53	60***	134	days of hospital re-admissions before and after baclofen initiation	average 19.1 days in the hospital per year compared to 25.48 days before treatment initiation			p 0.59

* post hoc analysis of Addolorato et al. 2007 (11)

** mean dose, range 30-210mg/d

*** median highest dose, 5-10 mg 5 times a day increased or decreased based on response and side effects
